# A Case Report of COVID-19 in New Orleans, Louisiana: Highlighting the Complexities of Prognostication in a Critically Ill Patient

**DOI:** 10.1089/pmr.2020.0087

**Published:** 2020-10-06

**Authors:** Zachary I. Lerner, Rebecca V. Burke, Jonathan McCall, Alexandra Leigh, Marcia Glass

**Affiliations:** ^1^ Louisiana State University School of Medicine, New Orleans, Louisiana, USA.; ^2^ Section of Geriatrics and Extended Care, Southeast Louisiana Veterans Health Care System (SLVHCS), New Orleans, Louisiana, USA.; ^3^ Department of Internal Medicine, Tulane School of Medicine, New Orleans, Louisiana, USA.

**Keywords:** COVID-19, intensive care, palliative care, prognostication

## Abstract

Palliative care teams and intensive care teams have experience providing goals-of-care guidance for critically ill patients and families. Critical coronavirus disease 2019 (COVID-19) infection is defined as infection requiring intensive care unit care, respiratory support, and often multiorgan involvement. This case presents a 53-year-old critically ill COVID-19 patient in multisystem organ failure who appeared hours from death despite best medical efforts. Comfort-focused care and compassionate extubation were offered after all medical teams felt near certain that death was imminent. Overnight, while options were being considered by the family, the patient began to markedly improve hemodynamically and was extubated several days later. Weeks later, the patient survived the hospital stay and was discharged to rehabilitation. After rehabilitation he returned home, able to walk, communicate freely, and independently perform all activities of daily living. Dialysis was no longer necessary and was stopped. The challenges of assisting in goals-of-care conversations for patients with serious COVID-19 infection are discussed.

## Introduction

Severe acute respiratory syndrome coronavirus 2 (SARS-CoV-2), the novel coronavirus responsible for coronavirus disease 2019 (COVID-19), was first identified in Wuhan, China, in January 2020 and has since spread globally, resulting in >26.1 million cases and 864,600 deaths worldwide as of September 2020.^1,2^

COVID-19 infection typically presents with fever, fatigue, and dry cough. Approximately 80% of infections are mild or asymptomatic, with minimal or absent lung involvement. Serious infection occurs in ∼15% of cases, characterized by dyspnea, tachypnea, hypoxemia, reduced PaO_2_ to FiO_2_ ratio, and extensive lung involvement. Critical infection occurs in ∼5% of cases, resulting in respiratory failure, septic shock, multiorgan dysfunction, and possibly death.^3,4^ Case fatality rates vary, due, in part, to differences in patient demographics, availability of testing, implementation of quarantine and contact tracing, and hospital resources.^5^ A case series of 72,314 COVID-19 patients in China showed a case fatality rate of 2.3% overall and 49.0% among critically ill patients.^4^

Furthermore, patients with comorbid hypertension, diabetes mellitus, cardiovascular disease, chronic lung disease, and cerebrovascular disease have an even higher risk of morbidity and mortality due to COVID-19; advanced age is also a risk factor for poor outcome.^3,6^ Complications of COVID-19 infection include bacterial superinfection, sepsis, multiorgan failure, disseminated intravascular coagulation (DIC), and acute respiratory distress syndrome (ARDS), all of which increase mortality.^3,7^ In COVID-19 patients with secondary infection, a mortality rate of 16% has been reported.^8^ COVID-19 patients with ARDS have a mortality rate as high as 28.8%.^9^ Of note, these complications independently increase risk of mortality; for instance, the mortality of patients with ARDS due to any cause is 40%.^10^

Ethnicity is now known to be an independent risk factor for increased mortality in COVID-19 patients. In one hospital in New Orleans, Louisiana, 76.9% of the patients who were hospitalized with COVID-19 and 70.6% of those who died were black, whereas blacks only comprised 31% of the hospital population.^11^

Many laboratory markers of disease severity have been studied, but a gold standard has yet to be identified as a prognostic marker or indicator of response to treatment. Elevated cardiac troponin, myoglobin, C-reactive protein, interleukin-6, and D-dimer have all been associated with an increased risk of death in patients with COVID-19.^8,12^

In this study we present a case of severe COVID-19 infection in a middle-aged patient with pre-existing comorbidities, followed by a discussion of the challenges of prognostication given our evolving understanding of this novel virus.

## Case Description

This is the case of a 53-year-old black male patient with a medical history of obesity, hypertension, hyperlipidemia, type 2 diabetes mellitus, and depression who presented to the emergency department (ED) in March of 2020 complaining of fever, cough, and worsening shortness of breath for one week. The patient had been caring for his mother who recently tested positive for COVID-19. He denied recent travel but endorsed intermittent headache and diarrhea. Home medications consisted of amlodipine 10 mg daily, carvedilol 25 mg twice daily, lisinopril 40 mg daily, atorvastatin 40 mg daily, and metformin 1000 mg twice daily. Social history was significant for recent discontinuance of tobacco and alcohol two months prior.

Vital signs in the ED included a temperature of 100.5° Fahrenheit (°F), blood pressure of 102/56 mmHg, pulse of 75 beats per minute, and oxygen saturation of 80%. Physical examination was positive for bilateral basilar crackles but otherwise normal. Complete blood count and basic metabolic panel were within normal limits. Chest X-ray showed patchy heterogeneous lower lung airspace opacities with findings representative of multifocal pneumonia. He was started on empiric treatment of community-acquired pneumonia with ceftriaxone and azithromycin while COVID-19 testing was pending and was admitted to the hospital on 4 L per minute of supplemental oxygen for further monitoring.

The patient's COVID-19 test returned positive and infectious disease recommended treatment with a five-day course of hydroxychloroquine. On day two of hospitalization, oxygenation was poor on 6 L per minute by nasal cannula, and the patient was escalated to a nonrebreather mask. Repeat chest X-ray showed increased bilateral lower lobe opacities, and by the evening the patient required endotracheal intubation and subsequent transfer to the intensive care unit (ICU) for treatment of ARDS.

Creatinine increased from 0.8 to 1.9 mg/dL. He subsequently became hypotensive and was started on norepinephrine for suspected cardiogenic shock. Blood and urine cultures returned negative. The ICU-prone ventilation protocol was initiated during this time to improve oxygenation to his lungs.

Renal function continued to decline, with creatinine rising overnight from 5.2 to 6.7 mg/dL, triggering a nephrology consult. One week into hospitalization, the patient developed ventilator-associated pneumonia and sputum culture grew *Pseudomonas aeruginosa*, which was treated with intravenous (IV) cefepime. The following day, he progressed to acute renal failure, necessitating continuous renal replacement therapy. Over the next several days, daily ventilator weaning was attempted unsuccessfully due to hypoxemia and tachypnea.

Two weeks after initial hospital admission, an inpatient palliative medicine consult was placed. At the time, the patient was septic with multiorgan failure, characterized by pulmonary, renal, cardiac, and possible neurologic compromise. Death seemed inevitable. Palliative medicine began engaging in discussions with the patient's family regarding goals of care, including offering palliative extubation and transition to comfort care. No decisions were immediately made, and the patient remained on all life-sustaining support. Palliative medicine continued to provide supportive counseling to the family.

Four days later, the patient developed atrial fibrillation with rapid ventricular response and an amiodarone infusion was started. The following day, the patient's right internal jugular central venous catheter (CVC) became occluded due to thrombosis. A femoral CVC was placed, which initially thrombosed as well despite heparin infusion. Repeat blood cultures remained positive for *P. aeruginosa*, and he was started on IV ciprofloxacin. Fortunately, the occluded femoral catheter remained a viable hemodialysis access site after troubleshooting. Laboratory tests suggested DIC as the etiology of thrombosis, with D-dimer 5.17 mcg/mL, fibrinogen 884 mg/dL, activated partial thromboplastin time 39.1 seconds, and C-reactive protein 14.6 mg/dL. Platelet count was normal.

Three days later, the patient began to follow commands and was successfully extubated that afternoon to high-flow nasal cannula. Several days after extubation, he remained obtunded and followed simple commands inconsistently, but was unable to engage in meaningful conversation. He experienced a transient fever of 102.7°F a few days later that resolved with removal of the femoral hemodialysis catheter.

Over the following several days, he began to recover his physical strength with intensive physical therapy, and his neurologic status improved significantly. He was transferred from the ICU to the medical/surgical ward almost one month after admission, where he continued to improve. His new-onset atrial fibrillation persisted, but his rate was successfully controlled. Cardiology service was consulted that recommended ablation. He underwent this procedure successfully and remained in normal sinus rhythm thereafter.

The patient was discharged to inpatient rehabilitation in May of 2020, where he remained for 10 days. He continued to require hemodialysis and was followed by the nephrology service as an outpatient. By June of 2020, his nephrologist determined that the patient had recovered enough renal function to discontinue hemodialysis. Despite such a dire inpatient hospital course, the patient eventually completely recovered physical strength, returned home, and is now walking, talking, and independent in all activities of daily living.

## Discussion

The COVID-19 pandemic has been a particularly challenging time for medical providers. As a critical component of the frontline medical workforce, palliative medicine providers have been called upon to help negotiate complex goals-of-care discussions and comfort grieving families. These situations can be difficult in the best of times and have proven to be particularly complex in recent months. Not only must medical providers stay up to date on evidence-based treatment protocols for a novel virus during a global pandemic, but we must also simultaneously balance the skill of comforting isolated patients with uncertain disease trajectories.

The case presented highlights the complexities of estimating prognosis solely utilizing laboratory-based and clinical indicators of severity. This is a black male with pre-existing comorbidities, which already placed him at high risk for mortality upon admission to the hospital with COVID-19; coupled with an ICU admission, ARDS, and multisystem organ failure, death appeared to be an inevitable outcome. Guided by limited data showing grim outcomes, his mortality upon entering the ICU ranged from 30% to 50%.^4,9^ Appropriately, the palliative medicine team strived to transparently communicate its concerns to the family that the patient may rapidly progress to death despite our best interventions. However, the body of medical knowledge pertaining to COVID-19 continues to evolve.

We now know that hydroxychloroquine does not reduce the risk of death in hospitalized COVID-19 patients,^13^ and alternatively steroids are of benefit in reducing mortality in severe cases.^14,15^ This ever-changing paradigm of how to treat COVID-19 patients can be exciting to some, but also perplexing and uncomfortable for hospital workers, family members, and patients.

Retrospectively, we question whether we were wrong to advise the family members of this patient to strongly consider palliative extubation. Although our motive was to prepare the family for what we genuinely believed to be inevitable demise, palliative extubation would have likely resulted in this patient's death.^16^ This is an uncomfortable reality we face as prognosticators for these critically ill patients. We have always provided recommendations guided by evidence-based medicine, but what if the evidence of poor outcomes in patients with comorbidities and complicated clinical course^3,8,9^ overshadows the instances of remarkable recoveries with this new virus?

Since caring for this patient, we provide a new disclaimer that predicting prognosis in COVID-19 patients is extremely difficult. We can advise based on the outcomes we have personally experienced in patients in similar predicaments, but conversely, we now clarify that we have seen miracles occur firsthand. Some may wonder whether this honesty weakens the confidence families will have in their providers, but from our experience treating more COVID-19 cases, it has in fact strengthened trust. This position is supported in a systematic review by LoCurto and Berg, who also conclude that honesty and communication are determinants of trust in a health care setting.^17^

Our COVID-19 patient exposure thus far has brought another concern to light that also hinders our ability to accurately predict prognosis: unconscious bias. Bias may influence how we process information about patients and lead to unintended health disparities. Evidence has shown that gender, age, and ethnicity bias among health care providers influences treatments offered to patients.^18^ Likewise, COVID-19 is unfortunately a virus associated with poorer outcomes in ethnic minorities, males, and elderly patients. This complicates bias-free prognostication even more.

In this case, we identify the availability heuristic and representativeness heuristic as likely sources of unconscious bias in predicting clinical course. Heuristics are problem-solving techniques commonly used by physicians to facilitate clinical decision making in the setting of limited time and incomplete information. Although useful, these techniques can introduce bias if improperly employed. In the availability heuristic, a previously -encountered diagnosis or clinical development that is more readily recalled by a clinician is considered to be more probable. This availability in recall is influenced by how recent or emotionally charged a memory is.^19^

During this patient's hospitalization in the setting of a pandemic, we treated other patients who ultimately died; the availability of these experiences for recall biased our decision making in treating this particular patient.

Similarly, the representativeness heuristic assesses probability of an outcome based on similarities between a case and a model.^20^ Although this method of pattern recognition is an important element of clinical decision making, overapplication can result in bias.^19^ In our case of a patient with severe COVID-19 complicated by multiple comorbidities, ICU admission, ARDS, and multiorgan failure, we incorrectly deemed this pattern to be representative of a terminal diagnosis.

From our experience, it is best to approach every COVID-19 patient, regardless of age, gender, race, or pre-existing comorbidities, as an individual with a unique response to treatment. In addition, we strive to retain awareness of the limitations of the heuristics we regularly employ as a part of our clinical decision making.

This case illustrates the inherent difficulties practicing medicine, especially palliative medicine, during the outbreak of a novel pandemic. Palliative teams are considered specialists in guiding families throughout the course of serious illness, with the expectation of ensuring patient and family understanding of prognosis. The unpredictable course of COVID-19 in each patient has exposed the magnitude of our recommendations in critically ill patients. Our suggestions may result in life or death. It is important for all of us to acknowledge our own fallibility, nurture our humility, and remain open to surprise outcomes and concomitant evolving decision making from families. As evidenced by this complicated case, death may seem imminent, but a complete turnaround is always possible.

## Abbreviations Used

ARDS: acute respiratory distress syndrome
COVID-19: coronavirus disease 2019
CVC: central venous catheter
DIC: disseminated intravascular coagulation
ED: emergency department
ICU: intensive care unit
IV: intravenous
SARS-CoV-2: severe acute respiratory syndrome coronavirus 2
